# Temperature imaging inside fluid devices using a ratiometric near infrared (NIR-II/III) fluorescent Y_2_O_3_: Nd^3+^, Yb^3+^, Er^3+^ nanothermometer

**DOI:** 10.1007/s44211-024-00564-0

**Published:** 2024-04-15

**Authors:** Masakazu Umezawa, Hikaru Haraguchi, Gaku Sugawara, Konosuke Sato, Hiroyuki Kurahashi, Teiji Oda, Kyohei Okubo, Kohei Soga

**Affiliations:** 1 Department of Materials Science and Technology, Faculty of Advanced Engineering, Tokyo University of Science, 6-3-1 Niijuku, Katsushika, Tokyo 125-8585 Japan; 2 Division of Cardiovascular Surgery, Department of Surgery, Shimane University Faculty of Medicine, 89-1 Enyacho, Izumo, Shimane 693-8501 Japan; 3 Present Address: Department of Materials Science and Engineering, School of Materials and Chemical Technology, Tokyo Institute of Technology, Nagatsuta-Cho 4259, Midori-Ku, Yokohama, Kanagawa 226-8503 Japan

**Keywords:** Rare-earth-doped ceramics, Imaging, Near-infrared, Fluorescence thermometry

## Abstract

Luminescence thermometry is a non-contact method that can measure surface temperatures and the temperature of the area where the fluorescent probe is located, allowing temperature distribution visualizations with a camera. Ratiometric fluorescence thermometry, which uses the intensity ratio of fluorescence peaks at two wavelengths with different fluorescence intensity dependencies, is an excellent method for visualizing temperature distributions independent of the fluorophore spatial concentration, excitation light intensity and absolute fluorescence intensity. Herein, Nd^3+^/Yb^3+^/Er^3+^-doped Y_2_O_3_ nanomaterials with a diameter of 200 nm were prepared as phosphors for temperature distribution measurement of fluids at different temperatures. The advantages of this designed fluorescent material include non-aggregation in water and the fact that its near-infrared (NIR) fluorescence excitation (808 nm) is not absorbed by water, thereby minimizing sample heating upon irradiation. Under optical excitation at 808 nm, the ratio of the fluorescence intensities of Yb^3+^ (I_Yb_; 975 nm) and Er^3+^ (I_Er_; 1550 nm), which exhibited different temperature responses, indicated the temperature distribution inside the fluid device. Thus, this technique using Nd^3+^/Yb^3+^/Er^3+^-doped Y_2_O_3_ is expected to be applied for temperature distribution mapping analysis inside fluidic devices as a ratiometric NIR fluorescence thermometer, which is unaffected by laser-induced heating.

**Graphical abstract:**

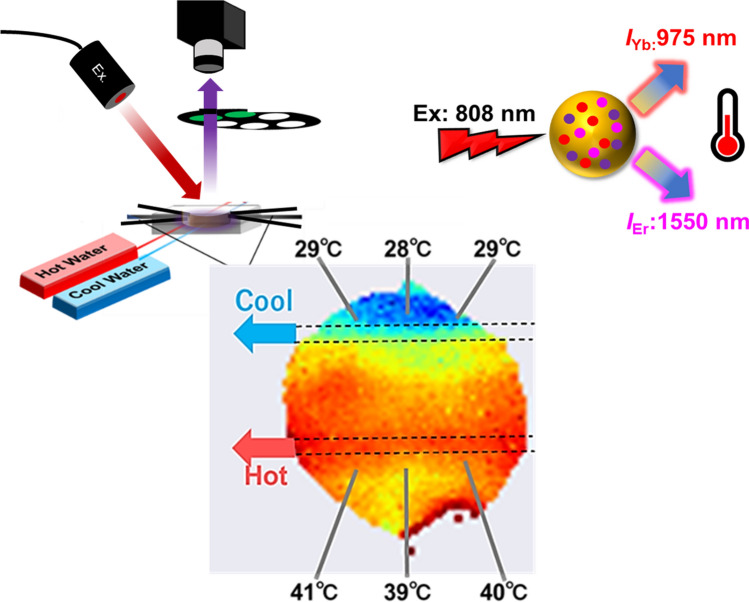

## Introduction

Thermography detecting mid-infrared radiation is often used to visualize temperature distributions, but the measurable temperature is limited to the surface temperature. Temperature measurements have a wide range of applications in automotive and aerospace engineering [1], chemistry [1], meteorology and oceanography [2, 3], medical biology [4, 5], electronics, production plants, and food storage [6]. In particular, the technical requirements for temperature measurements are increasing in various fields including photonics, nanoelectronics, nanofluidics, and biomedicine [6]; these requirements are expanding to the extent that conventional contact-type measurement methods such as thermocouples cannot satisfy the required submicron-scale spatial resolution. Fluorescence temperature measurements can be performed using contactless thermometry in microelectronics [7–10], microfluidic devices [9, 11–14], and catalytic systems [15]. Contactless temperature measurements using fluorescent particles have attracted considerable attention in the biomedical field for physiological temperature change measurements [16, 17]. Fluorescent materials, including chelated europium(III) [18–20], synthetic polymers [21, 22], and polymer nanoparticles [23–25], fluorescent proteins [26], and inorganic nanomaterials [27, 28] such as rare-earth-based phosphors [29] have been designed and developed for temperature measurements. However, the biomedical applications of most fluorescent materials operating in the ultraviolet and visible wavelength ranges are limited due to their lack of optical transparency in biological cells and tissues.

**Fig. 1:**
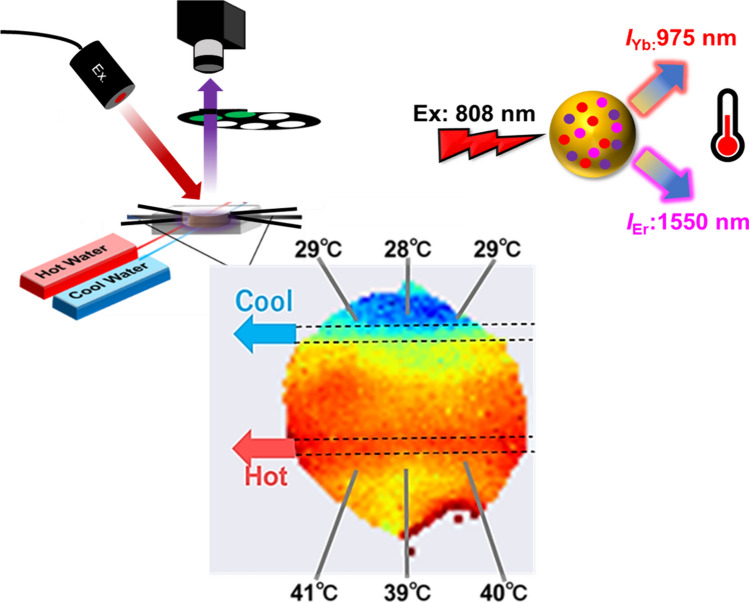
Two major principles of fluorescent thermometry. **a** Ratiometric fluorescence thermometry, **b** fluorescence lifetime-based thermometry

Near-infrared (NIR) light shows low optical loss in biological cells and tissues and is suitable for acquiring information in optically scattering structures [30]. Rare-earth-based phosphors function as major NIR fluorescent thermometers [31, 32] because their excitation-emission process involves temperature-sensitive multiphonon relaxation [33], thermal relaxation including vibrational and polaronic quenching [34], and phonon-assisted energy transfer [35, 36]. Thermometry utilizing the temperature dependence of various parameters of the luminescent centers of rare earth-based phosphors has been previously reported. Temperature can be measured by changes in the fluorescence intensity of a single or two wavelengths, fluorescence lifetime (Fig. 1) [37, 38], and spectral shifts, band shapes, and widths [6]. In particular, ratiometric NIR fluorescence thermometry, which uses the luminescence intensity ratio (LIR) of the peaks at two different wavelengths with different temperature dependences, has been reported as a self-referencing method for visualizing temperature distributions in the deep tissues, independent of the spatial concentration of fluorescent thermometers and excitation light intensity [16, 39–45]. This method has the advantage of easy preparation and experimental setup using two bandpass filters and a detector [46].

Herein, the target of the temperature measurement was a fluidic device of an artificial tissue, such as a lung model [47], which is a medical device that assists in adding oxygen to circulating blood. To maintain the efficiency of oxygen exchange and prevent denaturation of blood components, it is necessary to monitor the temperature distribution in the device. This study aimed to investigate a novel nanothermometry technique that enables temperature distribution visualization under physiological conditions and to demonstrate the performance of temperature imaging inside a fluidic device mimicking an artificial lung. Previously, β-NaYF_4_ nanoparticles co-doped with Yb^3+^, Ho^3+^, and Er^3+^ were reported as a type of simple rare-earth-doped ceramic without core–shell layers, which is applicable to ratiometric nanothermometry using LIR at 1150 nm (NIR-II) and 1550 nm (NIR-III) under excitation at 980 nm [40]. However, increases in the temperature of the target objects pose a problem during excitation owing to the minor absorption peak of water at 980 nm. Laser-induced heating can be minimized using a wavelength of 808 nm for excitation instead of 980 nm where water heating becomes a concern [48]. Nd^3+^ is widely used as a photosensitizer for 808 nm excitation, and combinations wherein Yb^3+^ bridges the energy transfer have been developed, such as in the Nd^3+^–Yb^3+^–Er^3+^ co-doped system [49]. In this study, we aimed to visualize the temperature distribution inside a fluidic device by ratiometric NIR-II/III fluorescence nanothermometry using Y_2_O_3_ nanoparticles co-doped with Nd^3+^, Yb^3+^, and Er^3+^ (Y_2_O_3_:Nd^3+^, Yb^3+^, Er^3+^) under 808 nm excitation. This method operates in the NIR region, where optical loss due to biological tissues is less; further, this method is expected to be applicable to samples containing blood in the flow.

## Results and discussion

**Fig. 2:**
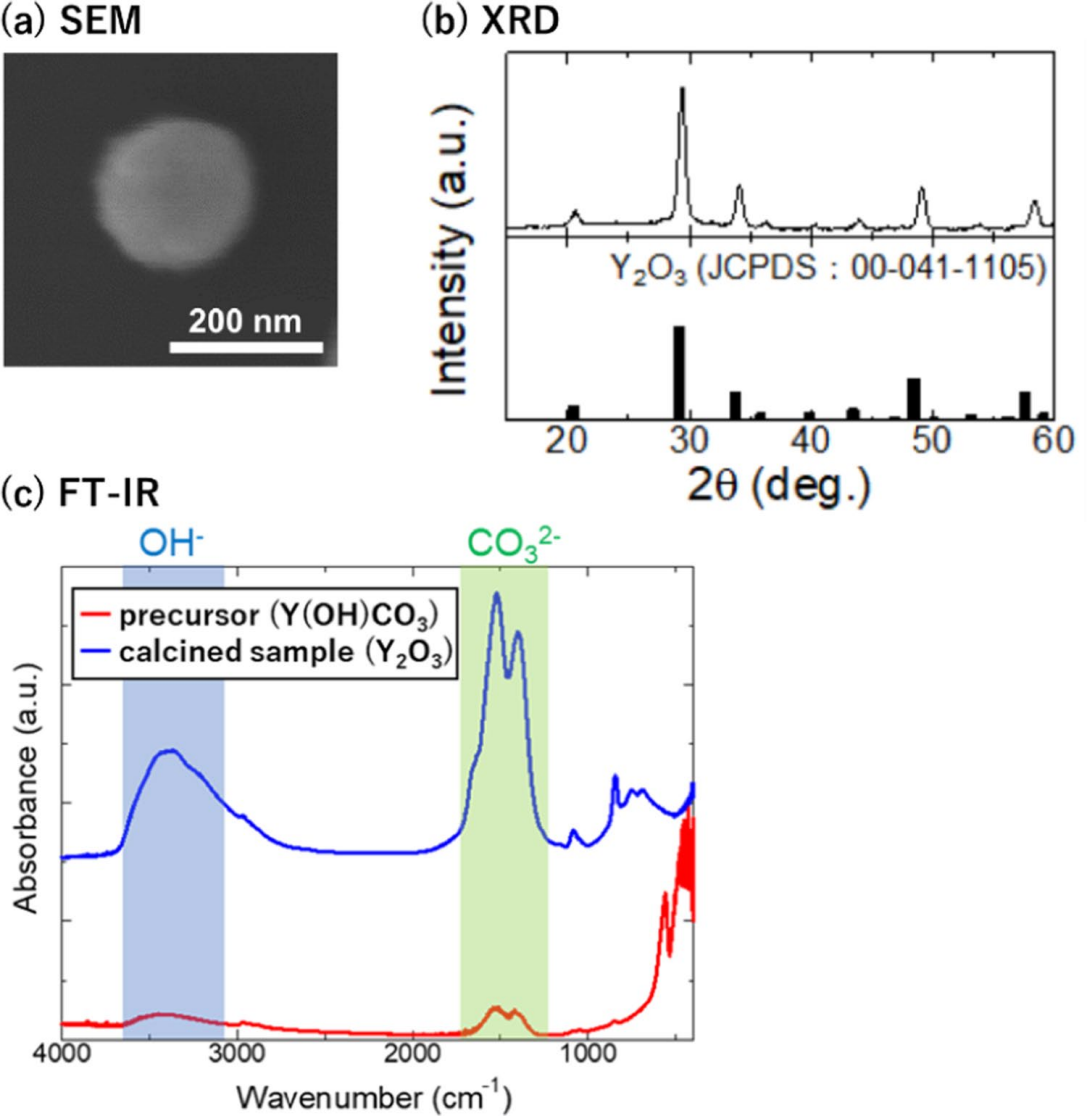
Characterization of the Y_2_O_3_:Nd^3+^, Yb^3+^, Er^3+^ nanoparticles. **a** Field emission scanning electron microscopic image of the synthesized Y_2_O_3_ nanoparticle. **b** X-ray diffraction patterns of the synthesized samples and Y_2_O_3_ crystal reference. **c** Fourier transform infrared spectra of the precursor and calcined samples

For application in the temperature imaging of artificial lung devices, a fluorescent nanothermometer with a particle size of approximately 200 nm synthesized in sufficient amounts was needed to ensure fluorescence intensity and perfusibility in fluidic devices. Y_2_O_3_ was selected as the host material to satisfy this requirement [50]. The precursor and calcined Y_2_O_3_:Nd^3+^, Yb^3+^, Er^3+^ synthesized using the homogeneous precipitation method [50] exhibited spherical shapes with a primary diameter of 200 nm (Fig. 2a, b). The X-ray diffraction pattern of the sample showed the typical characteristics of Y_2_O_3_ crystals (Fig. 2c). The Fourier transform infrared spectra showed that hydroxyl and carbonate groups were present in the precursor and were removed after calcination (Fig. 2d). Elemental analysis via inductively coupled plasma atomic emission spectroscopy showed that the calcined Y_2_O_3_:Nd^3+^, Yb^3+^, Er^3+^ samples contained Y^3+^, Nd^3+^, Yb^3+^, and Er^3+^ at 93.6, 0.86, 5.0, and 0.52 (mol%), respectively.

**Fig. 3:**
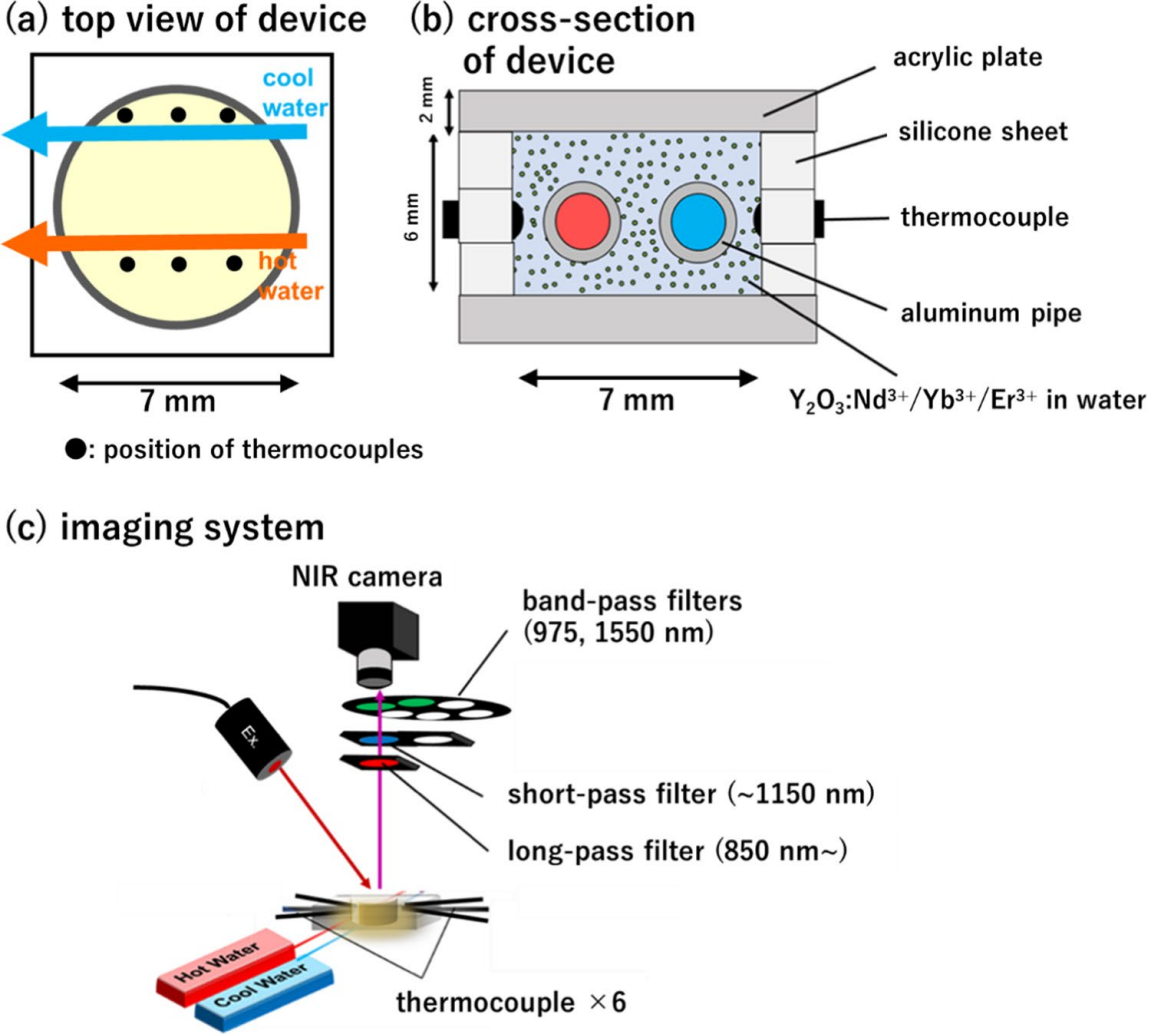
Experimental setup for temperature distribution imaging inside a fluid device. Schematic illustrations of **a** top view and **b** cross-sectional view of the fluid device prepared in this study. **c** Schematic illustration of the image acquisition system for different wavelength ranges in NIR

NIR fluorescence spectra (excitation wavelength: 808 nm) of the obtained samples were measured at 10–50 °C. As shown in Fig. 3a, Y_2_O_3_:Nd^3+^, Yb^3+^, Er^3+^ exhibited NIR fluorescence at 975 and 1030 nm (Yb^3+^: ^2^F_5/2_ → ^2^F_7/2_), 1060 nm (Nd^3+^: ^4^F_3/2_ → ^4^I_11/2_), and 1550 nm (Er^3+^: ^4^I_13/2_ → ^4^I_15/2_), respectively, under excitation at 808 nm (Nd^3+^: ^4^I_9/2_ → ^4^F_5/2_). Among these fluorescence peaks, the intensity at 975 nm (*I*_Yb_) decreased with increasing temperature, whereas that at 1550 nm (*I*_Er_) increased. As shown in the energy diagram (Fig. 3b), fluorescence at 975 and 1550 nm was emitted following a temperature-sensitive phonon-assisted energy transfer from Nd^3+^ (^4^F_3/2_) to Yb^3+^ (^2^F_5/2_) and Er^3+^ (^4^I_11/2_). The transfer between Yb^3+^ (^2^F_5/2_) and Er^3+^ (^4^I_11/2_) occurs via resonance energy transfer, which is not sensitive to temperature changes, whereas the thermal relaxation in Er^3+^ (from ^4^I_13/2_ to ^4^I_15/2_), emitting at 1550 nm, is enhanced by increasing temperature. This is the principle underlying the temperature-dependent increase in fluorescence at 1550 nm. In contrast, the temperature-dependent decrease in the fluorescence at 975 nm possibly occurred via the loss of phonon-assisted back transfer from Yb^3+^ (^2^H_5/2_) to Nd^3+^ (^4^F_3/2_). Thus, Y_2_O_3_:Nd^3+^, Yb^3+^, Er^3+^ NP operates as a fluorescent thermometer under excitation with 808 nm light to exhibit temperature-sensitive LIR (*I*_Er_/*I*_Yb_) that decreases with increasing temperature near the physiological temperature range (10–50 °C).

**Fig. 4:**
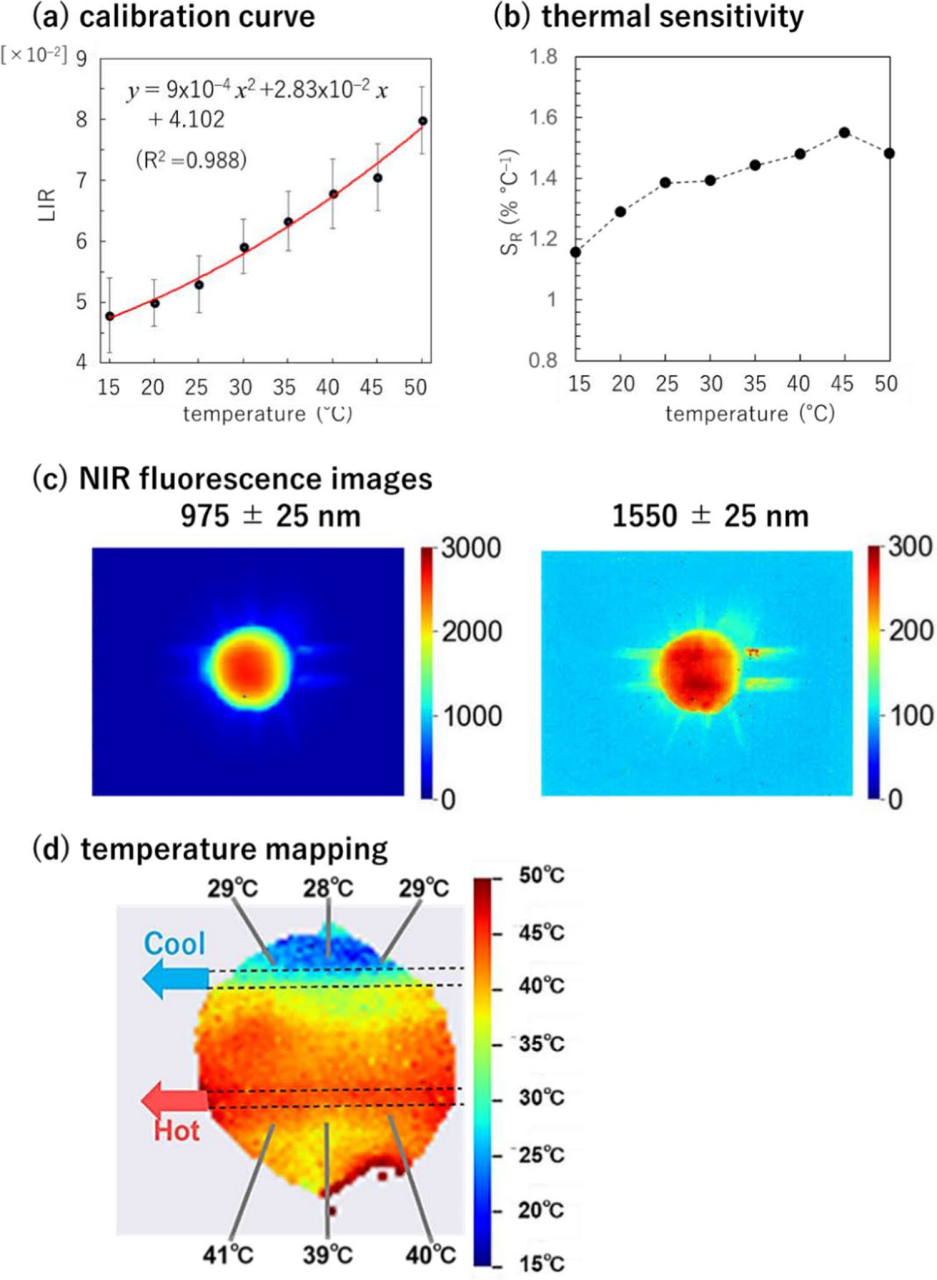
Temperature imaging of the fluidic device using the NIR fluorescence thermometer, Y_2_O_3_:Nd^3+^, Yb^3+^, Er^3+^ NPs (100 mg/mL). **a** The relation between temperature and luminescence intensity ratio (LIR = *I*_Er_/*I*_Yb_) of 975 (*I*_Yb_) and 1550 nm (*I*_Er_) on the images acquired with an NIR camera. Fluorescence images were acquired with integration time of 50 ms under 808 nm light excitation (260 mW/cm^2^). This data is used as a calibration curve to convert the fluorescence data to temperature values for each pixel. **b** Relative thermal sensitivity as functions of temperature for the Y_2_O_3_:Nd^3+^, Yb^3+^, Er^3+^ NPs. **c** Fluorescence intensity mapping of 975 ± 25 nm (*I*_Yb_) and 1550 ± 25 nm (*I*_Er_) in the fluidic device with an interior temperature gradient. **d** Measurement of the temperature distribution inside the fluidic device, while flowing hot and cold water in the two interior tubes by ratiometric fluorescence thermometry using LIR *I*_Er_/*I*_Yb_ based on the calibration curve in **a**. The reference temperatures were measured using a thermocouple inserted at each point

The prepared ratiometric fluorescence thermometer, Y_2_O_3_:Nd^3+^, Yb^3+^, Er^3+^ NPs, was used for temperature imaging inside a fluidic device, as shown in Fig. 3a, b. Two thin parallel tubes (diameter: 1 mm) that continuously flowed hot and cold water were passed through the device to create a temperature gradient. The interior was filled with water containing the dispersed Y_2_O_3_:Nd^3+^, Yb^3+^, Er^3+^ NPs. Under irradiation of 808 nm laser light, NIR fluorescence images were acquired by using an NIR camera with an InGaAs array detector through optical filters to collect images of fluorescence of 975 ± 25 nm (*I*_Yb_) and 1550 ± 25 nm (*I*_Er_) in the optical setup shown in Fig. 3c. Figure 4a shows the LIR calibration curve for Y_2_O_3_:Nd^3+^, Yb^3+^, Er^3+^ NPs dispersed in water as a function of temperature (15–50 °C). The LIR (*I*_Er_/*I*_Yb_) is positively correlated with temperature (Fig. 4a), while the relative thermal sensitivity, *S*_R_ = (1/LIR)∙d(LIR)⁄d*T* [16, 51], was 1.16% and 1.48% °C^−1^ at 15 and 50 °C, respectively (Fig. 4b), using *LIR* as the temperature-sensitive parameter.

NIR fluorescence images showing *I*_Yb_ and *I*_Er_ were acquired for the fluidic device through optical filters to select for the fluorescence wavelength ranges, where the temperature distribution was applied to the inside by flowing water of different temperatures into two parallel tubes. As shown in Fig. 4c, the fluorescence intensities of both wavelength bands were high at the center and decreased in the surrounding regions. This is because the excitation light intensity is not flat-topped but rather Gaussian distributed. Even in this case, the temperature can be measured by measuring the LIR in ratiometric fluorescent thermometry without being affected by the absolute fluorescence intensity. Therefore, temperature mapping was performed by converting LIR (*I*_Er_/*I*_Yb_) to temperature using a calibration curve (Fig. 4a) for each pixel (Fig. 4d). The accuracy of the ratiometric NIR fluorescence imaging was evaluated using the reference temperature data obtained by thermocouples inserted at six points in the fluidic device. The average difference between the reference temperature and the values measured by fluorescence thermometry was 1.5 °C. Despite this error, these results demonstrated that water-dispersed Y_2_O_3_:Nd^3+^, Yb^3+^, Er^3+^ nanoparticle are applicable for temperature distribution measurement inside the fluidic device under 808 nm excitation. High repeatability of thermometer readings has been reported for rare-earth-based inorganic luminescent thermometer [52, 53]. Although the repeatability is not examined for Y_2_O_3_:Nd^3+^, Yb^3+^, Er^3+^ NPs in this study, the successful visualization of the effects of heat and cool inside the fluidic devices also suggests a potential of high repeatability of our proposed thermometer.

## Materials and methods

### Materials

Y(NO_3_)_3_∙5H_2_O, Nd(NO_3_)_3_∙6H_2_O, Yb(NO_3_)_3_∙5H_2_O, and Er(NO_3_)_3_∙5H_2_O were purchased from Sigma-Aldrich (St. Louis, MO, USA). Urea was purchased from Kanto Chemical Co., Inc. (Tokyo, Japan). All reagents were used without further purification.

### Synthesis of Y_2_O_3_:Nd^3+^, Yb^3+^, Er^3+^

Y_2_O_3_:Nd^3+^, Yb^3+^, Er^3+^ nanoparticles were synthesized using the homogeneous precipitation method [50]. Briefly, Y(NO_3_)_3_∙5H_2_O (3.74 mmol), Nd(NO_3_)_3_∙6H_2_O (0.04 mmol), Yb(NO_3_)_3_∙5H_2_O (0.2 mmol), Er(NO_3_)_3_∙5H_2_O (0.02 mmol), and urea (1.2 mol) were mixed and dissolved in 200 mL of distilled water and heated at 90 °C for 1 h. After cooling to 20 °C, the precipitate was collected as a precursor by centrifugation (20,000*g*, 10 min) and washed with distilled water (20,000*g*, 10 min, twice). After drying the precursor at 80 °C for 24 h, it was calcinated by increasing the temperature to 600 °C by 50 min, further increased to 700 °C by 10 min, and maintained at 700 °C for 60 min in an electric furnace (NHK-170; Nitto Kagaku Co., Ltd., Nagoya, Japan) to yield Y_2_O_3_:Nd^3+^, Yb^3+^, Er^3+^ nanoparticles.

### Characterization of Y_2_O_3_:Nd^3+^, Yb^3+^, Er^3+^

The obtained samples were analyzed using X-ray diffraction (XRD; RINT-TTR III, Rigaku Co., Tokyo, Japan), Fourier transform infrared spectrometry (FT/IR-6000, JASCO Co., Tokyo, Japan), and scanning electron microscopy (SEM; S-4200, Hitachi High-Tech Co., Tokyo, Japan). The chemical composition was analyzed using an inductively coupled plasma atomic emission spectrometer (ICPE-9000, Shimadzu Co., Kyoto, Japan) for samples (3 mg) dissolved in aqua regia (1 mL) for 9 h and further diluted with distilled water (10 mL).

### Measurement of temperature-dependent change in near infrared fluorescence

NIR fluorescence spectra of the samples were collected using a fiber-coupled spectrometer (NIR Quest, Ocean Optics Inc., Dunedin, FL, USA) for the samples (100 mg/mL) dispersed in water under laser irradiation of 808 nm (260 mW/cm^2^). A fiber-coupled continuous-wave laser FL-FCSE08-7-808-200 (Focuslight Technologies Inc., Xi’an, China) was used as the excitation light source. The sample was held by a temperature-controllable holder (Qpod2e, Quantum Northwest, Inc., Liberty Lake, WA, USA) to obtain the NIR fluorescence data at different temperatures (10–50 °C). A long-pass filter (cut-off wavelength: 850 nm; #66-236, Edmond Optics, Inc., Barrington, NJ, USA) was placed between the sample and detector to prevent the excitation light from entering the detector.

### Preparation of fluid device with continuous fluid flow system

Three silicone sheets (thickness: 2 mm/sheet; K-125(50), Togawa Rubber Co., Ltd., Osaka, Japan) with a hollowed-out center were laminated and placed between two acrylic sheets (thickness: 2 mm/sheet; #2-9206-02, As One Co., Osaka, Japan) to create a fluidic device filled with a liquid resembling an artificial lung. To apply a temperature gradient to the liquid inside the device, two parallel flow paths of aluminum tubes (inner and outer diameters: 0.6 and 1 mm, respectively; #1426, Hikari Mall, Osaka, Japan), which could continuously pump hot and cold water, were passed through the fluid device (Fig. 2a). Six thermocouples (#3-1561-01, As One) were inserted for temperature monitoring.

### Ratiometric fluorescent temperature imaging

A schematic illustration of the image acquisition system for NIR fluorescence in the two wavelength ranges is shown in Fig. 2b. The NIR-II/III fluorescence images of the fluid device samples under light irradiation from an 808-nm laser diode were acquired using an NIR camera with a TE-cooled indium gallium arsenide (InGaAs) array detector (Xenics NV, Leuven, Belgium). A long-pass filter (cut-off wavelength: 850 nm; #66-236, Edmond Optics) was fixed between the sample and camera to prevent the excitation light from entering the detector. Bandpass filters for 975 ± 25 nm (#87-798, Edmond Optics) and 1550 ± 25 nm (#87-834, Edmond Optics) and a short-pass filter (cut-off wavelength: 1150 nm; #89-573, Edmond Optics) were used to select wavelength ranges to record for ratiometric temperature imaging.

## Conclusion

The NIR-II/III fluorescence intensity ratio, *I*_Er_/*I*_Yb_, of Y_2_O_3_:Nd^3+^, Yb^3+^, Er^3+^ under 808 nm excitation light showed a sensitive response to temperatures near room temperature (15–50 °C), suggesting utility for measuring temperature distribution in fluidic devices. Because 808 nm light is not absorbed by water, it can be applied to the temperature distribution mapping inside fluidic devices, such as artificial lungs, as a ratiometric NIR fluorescence thermometer that is unaffected by laser-induced heating. Because of the minimized optical scattering by biological membranes in the NIR-II/III region, the method presented herein is expected to be applicable even when the fluid in the device contains biological components, including red blood cells and other cells.

## Data Availability

The datasets used and analyzed during the current study are available from the corresponding authors on reasonable request.
